# Supplementary material for: Post-acute sequelae of COVID-19 is characterized by diminished peripheral CD8+β7 integrin+ T cells and anti-SARS-CoV-2 IgA response
Source: Nat Commun. 2023 Mar 30;14:1772. doi: 10.1038/s41467-023-37368-1 (PMC10061413; doi:10.1038/s41467-023-37368-1)
Supplement: Supplementary file 1 — Supplementary Information [file 41467_2023_37368_MOESM1_ESM.pdf]

Supplementary Information

Post-acute Sequelae of COVID-19 is Characterized by diminished peripheral $CD8^{+}\beta7$ $Integrin^{+}$ T cells and Anti-SARS-CoV-2 IgA response

Santa Cruz et al.

Supplementary Figure 1

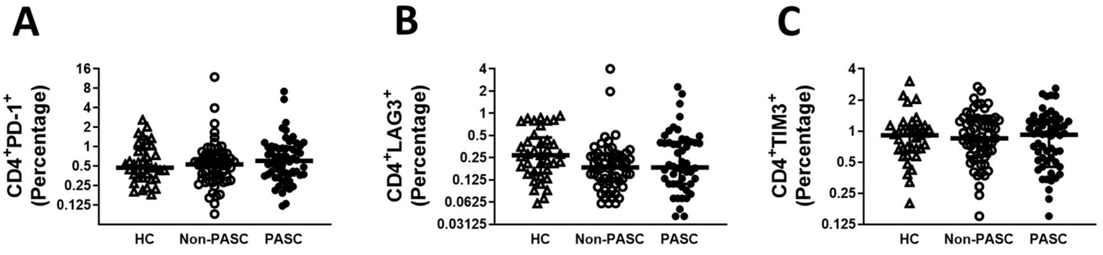

Supplementary Figure 1. CD4$^+$ T cell immune activation during SARS-CoV-2 infection on convalescents that developed PASC condition. (A) Percentages of the CD4$^+$ T lymphocytes expressing PD-1 (A), LAG3 (B) and TIM3 (C) of HC (n = 37), non-PASC (n = 65) and PASC (n = 62) individuals. Data are shown in a scatter dot plot format as median ± IQR

Supplementary Figure 2

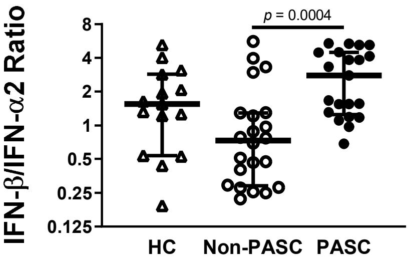

Supplementary Figure 2. CD4⁺ T cell immune activation during SARS-CoV-2 infection on convalescents that developed PASC condition. (A) Percentages of the $CD4^+$ T lymphocytes expressing PD-1 (A), LAG3 (B) and TIM3 (C) of HC (n = 37), non-PASC (n = 65) and PASC (n = 62) individuals. Data are shown in a scatter dot plot format as median ± IQR

Supplementary Figure 3

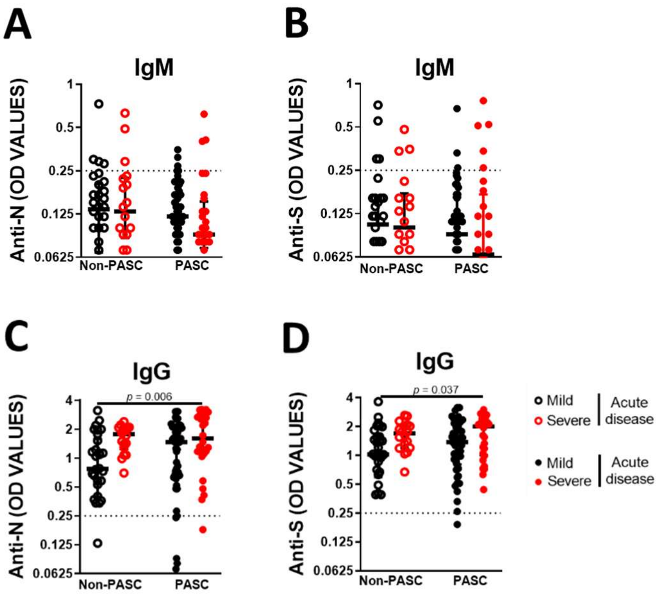

Supplementary Figure 3. Anti-N and anti-S humoral response. Quantification of the anti-N (A) and anti-S (B) IgM responses and anti-N (C) and anti-S (D) IgG in convalescent patients according to acute disease severity. Non-PASC individuals with mild (n = 30) or severe disease (n = 18) and PASC patients with mild (n = 50) and severe disease (n = 28) were tested. Data are shown in a scatter dot plot format as median ± IQR;

Supplementary Figure 4

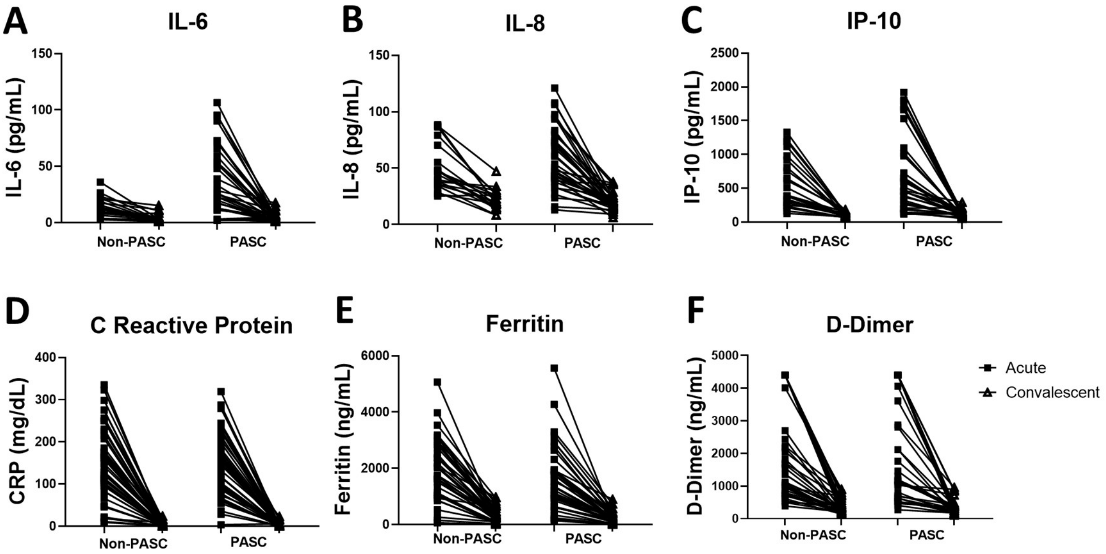

Supplementary Figure 4. Comparison of cytokine levels between acute disease and convalescence. The levels of IL-6 (A), IL-8 (B), IP-10 (C), CRP (D), ferritin (E), and d-dimer (F) were quantified on the plasma of acutely infected SARS-CoV2 (Acute) and at convalescence in individuals that develop (PASC) or do not develop (Non-PASC) post COVID-19 condition (Convalescent). For figures 6A-C, we tested non-PASC individuals during acute disease (n = 27) or convalescence (n = 29) and PASC patients during acute disease (n = 28) or convalescence (n = 30). For figures 6D-F, we tested non-PASC individuals during acute disease (n = 43) or convalescence (n = 41) and PASC patients during acute disease (n = 39) or convalescence (n = 36). Data are shown in a scatter dot plot format.

Supplementary Figure 5

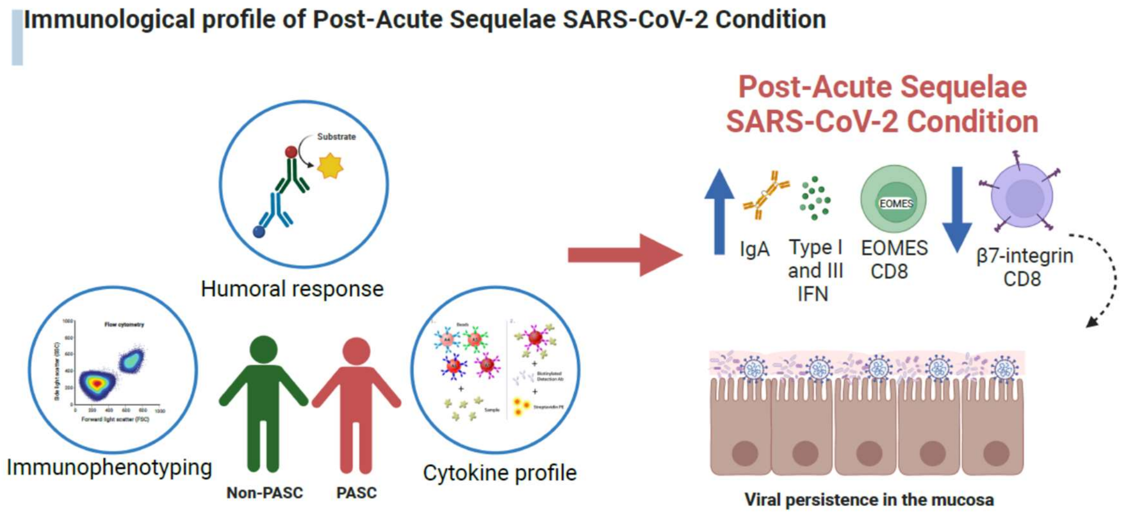

Supplementary Figure 5. Immunological profile of Post-Acute Sequelae of SARS-CoV-2 Condition (Created with BioRender.com)

Supplementary Figure 6

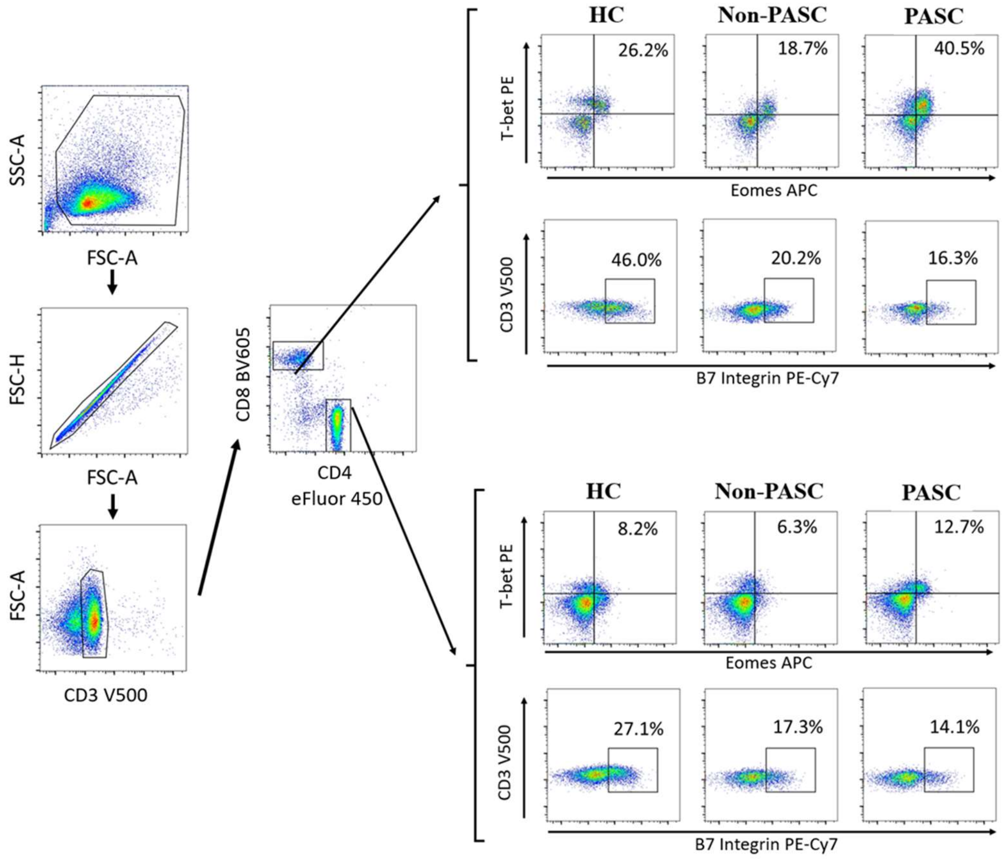

Supplementary Figure 6. Gating strategy. Gating strategy for Eomes^{+} T-bet^{+} CD4^{+} and CD8^{+} T lymphocytes as well as β7integrin^{+} CD4^{+} and CD8^{+} T lymphocytes.

Supplementary Figure 7

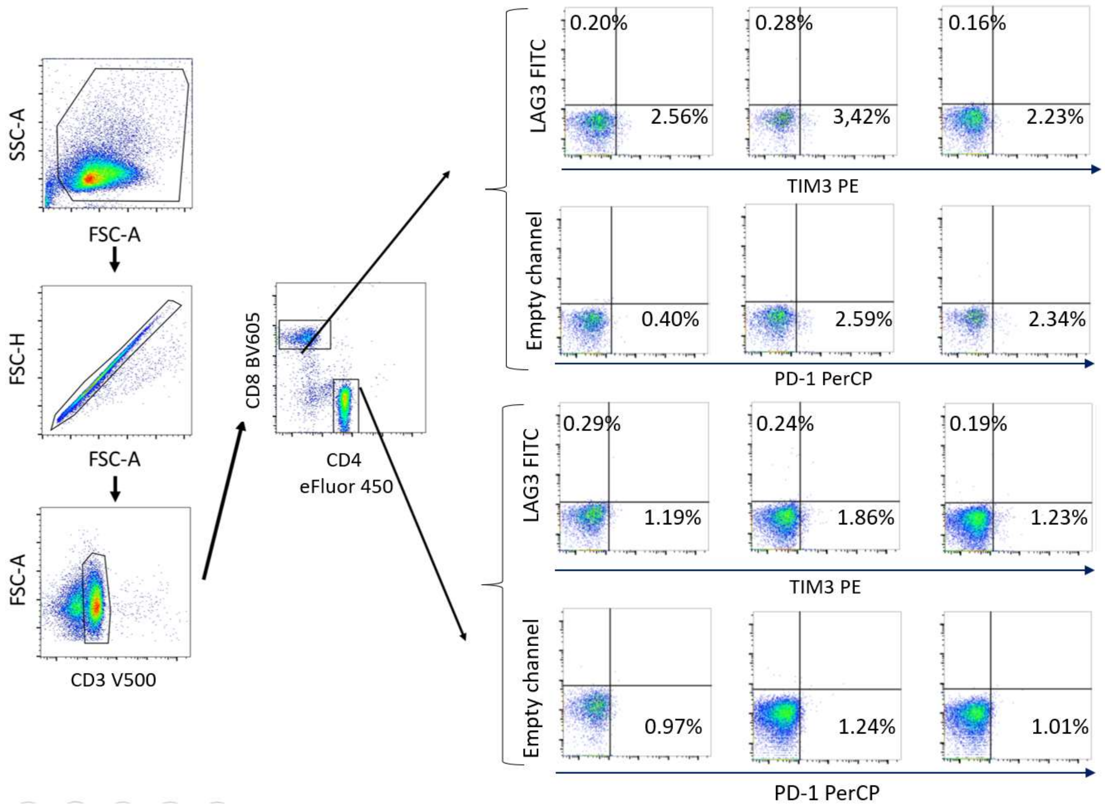

Supplementary Figure 7. Gating strategy. Gating strategy and representative histograms for PD-1^{+}, Lag-3^{+} and TIM-3^{+} CD4^{+} and CD8^{+} T lymphocytes.

Supplementary Figure 8

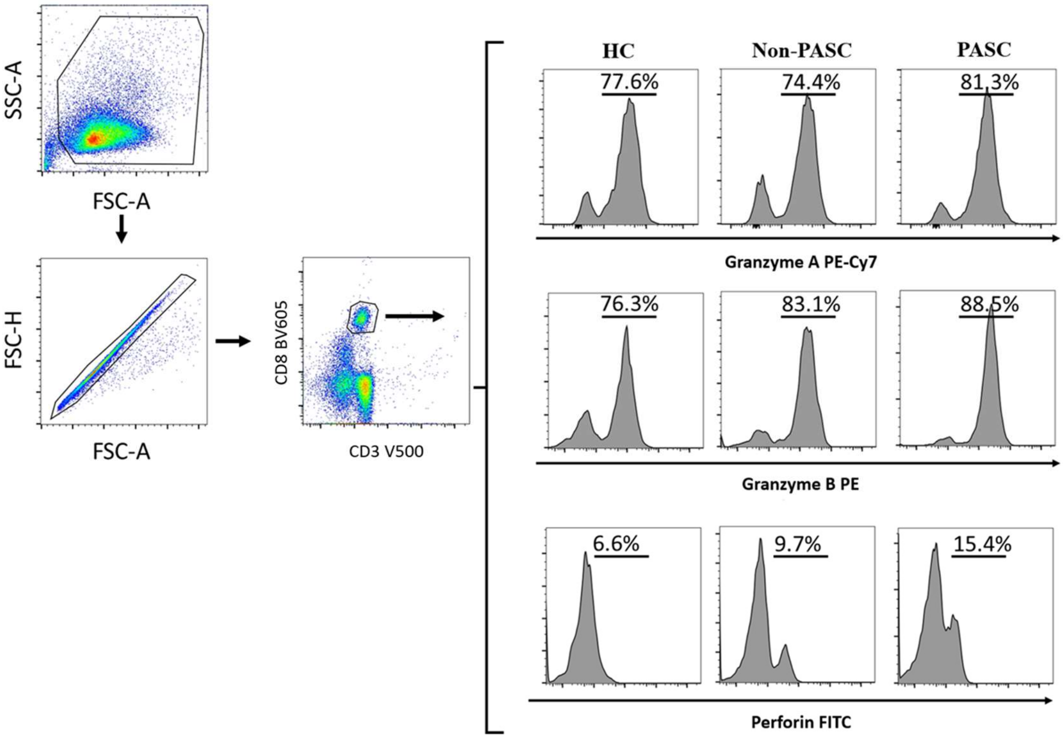

Supplementary Figure 8. Gating strategy. Gating strategy and representative histograms for the expression of granzyme A, granzyme B and Perforin on $CD8^{+}$ T lymphocytes.

Supplementary Table 1. Laboratory parameters evaluated during appointment six months after infection.

Parameter	Non-PASC (n=65)	PASC (n=62)	Mann-Whitney	
			r	*p*-value
**Laboratory parameters**, median (range)				
Lymphocyte count (lymphocyte/µL)	2100 (1300-4500)	2400 (1000-3600)	0.021	0.827
C-reactive Protein (mg/dL)	1.5 (0.5-23.7)	1.1 (0.5-96.8)	0.042	0.676
Ferritin (ng/mL)	261.0 (8.0-974.0)	181.0 (7.0-893.0)	0.140	0.166
D-dimers (ng/mL)	320.0 (127.0-1458.0)	360.0 (100.0-2183.0)	0.032	0.756

Supplementary Table 2: Comparison of inflammatory markers between acute and convalescent samples.

Variable	Df	F	*p*-value	Partial Eta Squared
**IL-6**	1, 69	0.009	0.92	0.001
**IL-8**	1, 70	0.742	0.39	0.010
**IP-10**	1, 70	0.634	0.43	0.009
**CRP**	1, 89	0.527	0.47	0.006
**Ferritin**	1, 81	0.319	0.57	0.004
**D-Dimers**	1, 82	0.080	0.78	0.001

A mixed-design ANOVA including PASC and non-PASC as between-subject factor and the pre-post measures as within-subject factors for all parameters to test the interaction between these two factors.

Supplementary Table 3: Antibody information.

Marker	Conjugated	Clone	Cat#	Manufacturer	Amount for 100µL
CD3	V500	SP34-2	560770	BD Biosciences	1.25
CD4	eFluor 450	OKT-4	48-0048-42	Invitrogen	1.25
CD8	BV605	SK1	344742	Biolegend	1.00
β7 integrin	PE-Cy7	FIB504	25-5867-42	Invitrogen	1.25
PD-1	PerCP	EH12.2H7	329938	Biolegend	1.25
LAG3	FITC	P18627	FAB2319F	R&D	2.50
TIM3	PE	344823	FAB2365P	R&D	1.25
Granzyme A	PE-Cy7	CB9	25-9177-42	Invitrogen	0.63
Tbet	PE	eBio4B10	12-5825-82	Invitrogen	1.25
Eomes	eFluor 660	WD1928	50-4877-42	Invitrogen	1.25
Granzyme B	PE	GB-11	M2289	Sanquin	0.63
Perforin	FITC	Pf-344	3465-7	MabTech	0.63
CD69	FITC	FN50	310904	Biolegend	4.00
Goat anti-Human IgG (Fc specific)[1]	Peroxidase	A0170	A0170	Millipore	0.02
Goat anti-Human IgM (Fc specific)[1]	Peroxidase	401905	401905	Millipore	0.02
Goat anti-Human IgA (Fc specific)[1]	Peroxidase	SAB3701229	SAB3701229	Millipore	0.02

Supplementary references:

[1] André S, Azarias da Silva M, Picard M, Alleaume-Buteau A, Kundura L, Cezar R, Soudaramourty C, André SC, Mendes-Frias A, Carvalho A, Capela C, Pedrosa J, Gil Castro A, Loubet P, Sotto A, Muller L, Lefrant JY, Roger C, Claret PG, Duvnjak S, Tran TA, Zghidi-Abouzid O, Nioche P, Silvestre R, Corbeau P, Mammano F, Estaquier J. Low quantity and quality of anti-spike humoral response is linked to CD4 T-cell apoptosis in COVID-19 patients. Cell Death Dis. 2022 Aug 27;13(8):741.
