# Supplementary material for: Post-acute sequelae of COVID-19 is characterized by diminished peripheral CD8+β7 integrin+ T cells and anti-SARS-CoV-2 IgA response
Source: Nat Commun. 2023 Mar 30;14:1772. doi: 10.1038/s41467-023-37368-1 (PMC10061413; doi:10.1038/s41467-023-37368-1)
Supplement: Supplementary file 3 — Reporting Summary [file 41467_2023_37368_MOESM3_ESM.pdf]

nature portfolio

Corresponding author(s): Ricardo Silvestre

Last updated by author(s): Feb 28, 2023

Reporting Summary

Nature Portfolio wishes to improve the reproducibility of the work that we publish. This form provides structure for consistency and transparency in reporting. For further information on Nature Portfolio policies, see our Editorial Policies and the Editorial Policy Checklist.

Statistics

For all statistical analyses, confirm that the following items are present in the figure legend, table legend, main text, or Methods section.

n/a	Confirmed	
☐	☒	The exact sample size (*n*) for each experimental group/condition, given as a discrete number and unit of measurement
☒	☐	A statement on whether measurements were taken from distinct samples or whether the same sample was measured repeatedly
☐	☒	The statistical test(s) used AND whether they are one- or two-sided<br>*Only common tests should be described solely by name; describe more complex techniques in the Methods section.*
☒	☐	A description of all covariates tested
☐	☒	A description of any assumptions or corrections, such as tests of normality and adjustment for multiple comparisons
☐	☒	A full description of the statistical parameters including central tendency (e.g. means) or other basic estimates (e.g. regression coefficient) AND variation (e.g. standard deviation) or associated estimates of uncertainty (e.g. confidence intervals)
☐	☒	For null hypothesis testing, the test statistic (e.g. *F*, *t*, *r*) with confidence intervals, effect sizes, degrees of freedom and *P* value noted<br>*Give P values as exact values whenever suitable.*
☒	☐	For Bayesian analysis, information on the choice of priors and Markov chain Monte Carlo settings
☒	☐	For hierarchical and complex designs, identification of the appropriate level for tests and full reporting of outcomes
☒	☐	Estimates of effect sizes (e.g. Cohen's *d*, Pearson's *r*), indicating how they were calculated

Our web collection on statistics for biologists contains articles on many of the points above.

Software and code

Policy information about availability of computer code

Data collection	BD FACSDiva Software version 6.1.3
Data analysis	All statistical analysis were done on SPSS version 28 software and data was plotted using Graphpad version 8.4.2.<br>FACS data was analized using LEGENDplex™analysis software (web version: https://legendplex.qognit.com/user/login?next=home) and FlowJo software v10.8.1.

For manuscripts utilizing custom algorithms or software that are central to the research but not yet described in published literature, software must be made available to editors and reviewers. We strongly encourage code deposition in a community repository (e.g. GitHub). See the Nature Portfolio guidelines for submitting code & software for further information.

Data

Policy information about availability of data

All manuscripts must include a data availability statement. This statement should provide the following information, where applicable:

- Accession codes, unique identifiers, or web links for publicly available datasets
- A description of any restrictions on data availability
- For clinical datasets or third party data, please ensure that the statement adheres to our policy

The data generated in this study are provided in the Source Data file. Patients-related data were generated as part of clinical examination and may be subject to donor confidentiality.

Human research participants

Policy information about studies involving human research participants and Sex and Gender in Research.

Reporting on sex and gender	Both male and female patients could be equally recruited, as study design intended to generate a sample that could reflect the patient population without any bias, even if a predominance of male patients was anticipated according to data from other studies. Information regarding patients' sex was collected as to all the other variables used. We could not find any influence of sex on our results, which means our findings apply to both sexes.
Population characteristics	A total of 127 patients were included, with a median age of 63 years (minimum 24 and maximum 87), with 67% of patients being male. Information regarding major comorbidities, severity of disease, level of support, common laboratory parametersand reported symptoms were all collected and are described in table 1.
Recruitment	Patients could be recruited at admission due to acute disease or at the outpatient setting, during a follow-up consultation.Only patients able to give their informed consent could be recruited. Further inclusion and exclusion criteria are clearlystated in the manuscript. Due to work overload and limited resources, it was not possible to invite all inpatients toparticipate. However, we can not identify any specific bias in the process that may significantly influence the results.
Ethics oversight	In respect to the Portuguese Law 21/2014, our research complies with the local Ethics Committee of Braga Hospital that approved the study with the reference 123/2020 (approved on 09/09/2020). Volunteering participants gave written informed consent in compliance with the Declaration of Helsinki principles.

Note that full information on the approval of the study protocol must also be provided in the manuscript.

Field-specific reporting

Please select the one below that is the best fit for your research. If you are not sure, read the appropriate sections before making your selection.

☒ Life sciences ☐ Behavioural & social sciences ☐ Ecological, evolutionary & environmental sciences

For a reference copy of the document with all sections, see nature.com/documents/nr-reporting-summary-flat.pdf

Life sciences study design

All studies must disclose on these points even when the disclosure is negative.

Sample size	No minimum sample size calculation was performed as this was an exploratory study intending to describe a broad collection of immunemechanisms that can be altered in a new disease. We understand that the final sample size is enough considering that all sub-groups of age,sex, disease severity and support are represented by a reasonable number of individuals. Also, we also could find robust tendencies andsignificant results with our sample size. By adding a control group with a dimension that is generally accepted, we also add reliability to ourresults
Data exclusions	No data were excluded from the analysis.
Replication	Sample constitution and clinical information gathering was the most complete and accurate possible. All the assays involved testing inindependent clinical samples from 127 patients and controls. All the assays were performed once with two technical replicates and withappropriate secondary reference controls. All duplicated experiments were sucessful and we still have more samples left if a retest isnecessary.
Randomization	This study is not a clinical trial. Therefore, randomization is not applicable. The groups were established according to the clinical status of the patients and the analysis was performed on that basis.
Blinding	The Investigators were blinded to group allocation during data collection and analysis. A code was assigned to each participant and the datacollection and analysis was performed without the knowledge of which group they appertain.

Reporting for specific materials, systems and methods

We require information from authors about some types of materials, experimental systems and methods used in many studies. Here, indicate whether each material, system or method listed is relevant to your study. If you are not sure if a list item applies to your research, read the appropriate section before selecting a response.

Materials & experimental systems

n/a	Involved in the study
☐	☒ Antibodies
☒	☐ Eukaryotic cell lines
☒	☐ Palaeontology and archaeology
☒	☐ Animals and other organisms
☒	☐ Clinical data
☒	☐ Dual use research of concern

Methods

n/a	Involved in the study
☒	☐ ChIP-seq
☐	☒ Flow cytometry
☒	☐ MRI-based neuroimaging

Antibodies

Antibodies used

1-CD3 V500 - clone: SP34-2, cat. 560770, BD Biosciences
2-CD4 eFluor 450 - clone: OKT-4, cat. 48-0048-42, Invitrogen
3-CD8 BV605 - clone: SK1, cat. 344742, Biolegend
4-β7 integrin PE-Cy7, clone: FIB504. cat. 25-5867-42, Invitrogen
5-PD-1 PerCP, clone: EH12.2H7, cat. 329938, Biolegend
6-LAG3 FITC, clone: P18627, cat. FAB2319F, R&D
7-TIM3 PE, clone: 344823, cat. FAB2365P, R&D
8-Granzyme A PE-Cy7, clone: CB9, cat. 25-9177-42, Invitrogen
9-Tbet PE, clone: eBio4B10, cat. 12-5825-82, Invitrogen
10-Eomes eFluor 660, clone: WD1928, cat. 50-4877-42, Invitrogen
11-Granzyme B PE, clone: GB-11, cat. M2289, Sanquin
12-Perforin FITC, clone: Pf-344, cat. 3465-7, MabTech
13-CD69 FITC, clone: FN50, cat. 310904, Biolegend
14-Goat anti-Human IgG (Fc specific)1 Peroxidase, clone: A0170, cat. A0170, Millipore
15-Goat anti-Human IgM (Fc specific)1 Peroxidase, clone: 401905, cat. 401905, Millipore
16-Goat anti-Human IgA (Fc specific)1 Peroxidase, clone: SAB3701229, cat. SAB3701229, Millipore

Validation

1- https://www.bdbiosciences.com/en-us/products/reagents/flow-cytometry-reagents/research-reagents/single-color-antibodies-ruo/v500-mouse-anti-human-cd3.560770
2- https://www.thermofisher.com/antibody/product/CD4-Antibody-clone-OKT4-OKT-4-Monoclonal/48-0048-42
3- https://www.biolegend.com/fr-lu/products/brilliant-violet-605-anti-human-cd8-antibody-12406
4- https://www.thermofisher.com/antibody/product/Integrin-beta-7-Antibody-clone-FIB504-Monoclonal/25-5867-42
5- https://www.biolegend.com/en-us/products/percp-anti-human-cd279-pd-1-antibody-9865
6- https://www.rndsystems.com/products/human-lag-3-fluorescein-conjugated-antibody_fab2319f
7- https://www.rndsystems.com/products/human-tim-3-pe-conjugated-antibody-344823_fab2365p
8- https://www.thermofisher.com/antibody/product/Granzyme-A-Antibody-clone-CB9-Monoclonal/25-9177-42
9- https://www.thermofisher.com/antibody/product/T-bet-Antibody-clone-eBio4B10-4B10-Monoclonal/12-5825-82
10- https://www.thermofisher.com/antibody/product/EOMES-Antibody-clone-WD1928-Monoclonal/50-4877-42
11- https://www.sanquin.org/products-and-services/reagents/products/immune-reagents/cytokines/m2289
12- https://www.mabtech.com/products/anti-human-perforin-antibody-pf-344-fitc-3465-7-0
13- https://www.biolegend.com/en-us/products/fitc-anti-human-cd69-antibody-1671?GroupID=BLG10036
14- https://www.sigmaaldrich.com/PT/en/product/sigma/a0170
15- https://www.merckmillipore.com/PT/en/product/Goat-Anti-Human-IgM-Chain-Specific-Peroxidase-Conjugate,EMD_BIO-401905?ReferrerURL=https%3A%2F%2Fwww.google.com%2F
16- https://www.sigmaaldrich.com/PT/en/product/sigma/sab3701229

Flow Cytometry

Plots

Confirm that:

☒ The axis labels state the marker and fluorochrome used (e.g. CD4-FITC).

☒ The axis scales are clearly visible. Include numbers along axes only for bottom left plot of group (a 'group' is an analysis of identical markers).

☒ All plots are contour plots with outliers or pseudocolor plots.

☒ A numerical value for number of cells or percentage (with statistics) is provided.

Methodology

Sample preparation	Blood samples were collected using Heparin Blood Collection tubes (VACUETTE). After transportation to ICVS, samples wereprocessed in BSL2 laboratories. Peripheral blood mononuclear cells (PBMCs) were isolated using equal volumes of peripheralblood and Histopaque 1077 (MilliporeSigma, St Louis, Missouri, USA). After centrifugation, plasma samples were collectedand stored (-80°C). PBMCs were frozen in Fetal Bovine Serum (FBS, Gibco, Thermo Fisher Scientific) with 10% of DMSO.PBMCs were thawed and divided in two 96-well plates for immune phenotyping. Surface staining was performed with theabove mentioned antibodies, followed by an intracelular staining using the Foxp3 / Transcription Factor Staining Buffer Set(Ref. LTI 00-5523-00, Invitrogen).
Instrument	LSRII flow cytometer (BD Biosciences)
Software	Samples were acquired using the BD FACSDiva software V6.1.3 and data was analyzed using FlowJo software.
Cell population abundance	In all experiments, at least 10.000 CD4 or CD8 T cells were analysed.
Gating strategy	Lymphocyte popolation was chosen first (SSC vs FSC), then singlets (FSC-H vs FSC), then CD3 positive cells were isolated toget CD4 and CD8 cells. The gating strategy is included in the manuscript as requested.

☒ Tick this box to confirm that a figure exemplifying the gating strategy is provided in the Supplementary Information.
